# Fecal source identification using random forest

**DOI:** 10.1186/s40168-018-0568-3

**Published:** 2018-10-18

**Authors:** Adélaïde Roguet, A. Murat Eren, Ryan J Newton, Sandra L McLellan

**Affiliations:** 1 School of Freshwater Sciences, University of Wisconsin-Milwaukee, Milwaukee, WI USA; 2 Department of Medicine, University of Chicago, Chicago, IL USA

**Keywords:** Microbial source tracking, 16S rRNA gene, High-throughput sequencing, *Clostridiales*, *Bacteroidales*, Random forest classification

## Abstract

**Background:**

*Clostridiales* and *Bacteroidales* are uniquely adapted to the gut environment and have co-evolved with their hosts resulting in convergent microbiome patterns within mammalian species. As a result, members of *Clostridiales* and *Bacteroidales* are particularly suitable for identifying sources of fecal contamination in environmental samples. However, a comprehensive evaluation of their predictive power and development of computational approaches is lacking. Given the global public health concern for waterborne disease, accurate identification of fecal pollution sources is essential for effective risk assessment and management. Here, we use random forest algorithm and 16S rRNA gene amplicon sequences assigned to *Clostridiales* and *Bacteroidales* to identify common fecal pollution sources. We benchmarked the accuracy, consistency, and sensitivity of our classification approach using fecal, environmental, and artificial in silico generated samples.

**Results:**

*Clostridiales* and *Bacteroidales* classifiers were composed mainly of sequences that displayed differential distributions (host-preferred) among sewage, cow, deer, pig, cat, and dog sources. Each classifier correctly identified human and individual animal sources in approximately 90% of the fecal and environmental samples tested. Misclassifications resulted mostly from false-positive identification of cat and dog fecal signatures in host animals not used to build the classifiers, suggesting characterization of additional animals would improve accuracy. Random forest predictions were highly reproducible, reflecting the consistency of the bacterial signatures within each of the animal and sewage sources. Using in silico generated samples, we could detect fecal bacterial signatures when the source dataset accounted for as little as ~ 0.5% of the assemblage, with ~ 0.04% of the sequences matching the classifiers. Finally, we developed a proxy to estimate proportions among sources, which allowed us to determine which sources contribute the most to observed fecal pollution.

**Conclusion:**

Random forest classification with 16S rRNA gene amplicons offers a rapid, sensitive, and accurate solution for identifying host microbial signatures to detect human and animal fecal contamination in environmental samples.

**Electronic supplementary material:**

The online version of this article (10.1186/s40168-018-0568-3) contains supplementary material, which is available to authorized users.

## Background

In urban areas downstream from mixed-land use watersheds, fecal contamination originates from multiple sources including sewage released from pipe-infrastructure, upstream agricultural animals, domestic pets, and/or wildlife. Identifying the contamination source is critical for managing public health risk, but sorting out pollutant contributors is difficult. The specific architecture of the gut microbiome in humans and animals could be useful for this purpose. As a result of the co-evolution between hosts and their gut microbiota driven by shared dietary regimes or host physiology [1–4], hosts have non-random and distinct gut microbial community structures [5–7]. These differences can serve as signatures for fecal sources in environmental samples with complex microbial community mixtures [8].

Monitoring for traditional fecal indicator bacteria such as *Escherichia coli* and enterococci does not provide information about contamination sources as these indicators are found indiscriminately in warm-blooded animals [9]. Yet, source information is essential for risk mitigation since gastrointestinal illnesses associated with exposure to contaminated waters can vary according to the host source and the pathogens they harbor. For example, the risks associated with human or cattle feces appear to be higher than with pig or avian feces [10, 11].

Multiple studies have used high-throughput sequencing of microbial community composition and advanced computational approaches to identify contamination sources in different environments, including indoor habitats [12–15] and surface waters [6, 7, 16–20]. SourceTracker [12], a state-of-the-art Bayesian classifier, is the primary platform that has been used to determine microbial source contamination in mixed-assemblage or “sink” environmental samples [6, 7, 16–20]. Although SourceTracker could be used for fecal source identification, each new investigation requires all source and sink samples of interest to be re-analyzed de novo. This setup requires investigators to either generate microbial source (e.g., human and animal fecal samples) sequence data or mine databases for appropriate information to pair with their environmental samples, decreasing its feasibility to be used widely. We propose an alternative approach to classify fecal sources using random forest. Random forest is one of the most accurate learning algorithms for handling large and noisy datasets [21]. Unlike SourceTracker, the classifiers generated by random forest can be used later to rapidly classify new data rather than re-analyzing the entire dataset. Random forest is also a model that can handle unbalanced sample distributions and is less prone to overfitting, which produces unbiased classifiers [22]. This machine learning approach has been used to classify body site, subject, and diagnoses using human microbiome datasets [23], but performance has not been evaluated for fecal source identification purposes.

The majority of source identification studies examine the entire bacterial community structure [6, 7, 16–20]. However, focusing on specific taxa may be sufficient for predictions [24, 25]. The two bacterial groups *Clostridiales* and *Bacteroidales* are ideal targets since they are highly abundant in the gastrointestinal tract of animals and humans [4], and contain members that show distinct host distribution patterns [8]. In this study, we developed a random forest-based classification approach to perform fecal source identification using microbial community data. We first built reference sequence databases for eight source categories using amplicon sequences generated from the V6 and V4V5 regions of the 16S rRNA gene: human (sewage), cat, cow, dog, deer, pig, pet (cat and dog), and ruminant (cow and deer). Using these fecal source samples, environmental water samples, and in silico artificial assemblages, we then evaluated the performance of *Bacteroidales* and *Clostridiales* classifiers to identify fecal contamination sources. Our benchmarks included assessment of prediction accuracy, repeatability, and sensitivity of each classifier for each animal group.

## Methods

### Random forest application to source tracking

#### Theory

Random forest, developed by Breiman [26], is an ensemble learning method, i.e., a strategy that aggregates many predictions to reduce the variance and improve robustness and precision of outputs [27]. Particularly well adapted to perform classification analysis [28], this powerful statistical approach has been used successfully in a wide range of applications, including source identification using PCR markers for fecal pollution and antibiotic resistance profiles [29, 30]. This approach appears to be suitable for source identification using sequence data because it can (i) relatively quickly analyze large datasets, e.g., sequencing datasets, (ii) provide very high classification accuracy of source, and (iii) estimate the importance for each predictor, i.e., representative sequence.

Random forest classification algorithm is based on the construction of multiple decision trees according to the bagging approach: each tree is constructed independently from a bootstrap sample of the entire dataset. In addition, to avoid overfitting of the model, each decision point, so-called node, is split using the best abundance threshold among a subset of predictors randomly selected rather than using the best among all representative sequences. The best split is based on the Gini criterion, which for each node decreases impurity as much as trees grow. To classify an unknown sample, new data are mapped along the trees built to train the model. Each tree gives a classification, so-called the voting tree. The majority votes among all the tree outcomes are taken to assess the prediction.

To evaluate the accuracy of classifications, an error rate of the global prediction, so-called “out-of-bag error rate”, is estimated. For that, during tree growth, the bootstrap sample repetition omits about one-third of the training samples, constituting the out-of-bag samples. Considered as new unknown samples, out-of-bag samples are classified among the different sources using the majority vote. For each model, a misclassification rate, i.e., out-of-bag error rate, is assessed by aggregating the cross-validation results between the predictions and the true sources.

The mean decrease in Gini value identifies the most reliable and relevant predictors to perform classifications. One value is associated with each individual representative sequence, reflecting the reliability to split the nodes. It is calculated by summarizing all the decreases in impurity scores associated with a given representative sequence, which is then normalized by the number of trees.

#### Source identification application

**Fig. 1:**
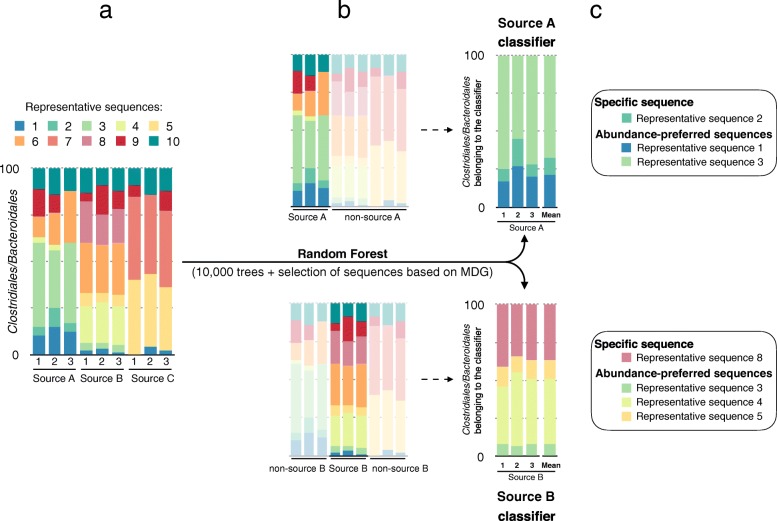
Design of the classifiers. (**a**) Determination of the bacterial group profiles for each of the source samples. (**b**) Analysis of the bacterial profile discrepancies between the samples that belong to the targeted source and all other samples using random forest (one classifier per source). (**c**) Selection of the relevant representative sequences based on mean decrease Gini values to form the classifier. Classifiers are then trained using the selected representative sequences. *Abbreviations*: *MDG* mean decrease Gini

**Fig. 2:**
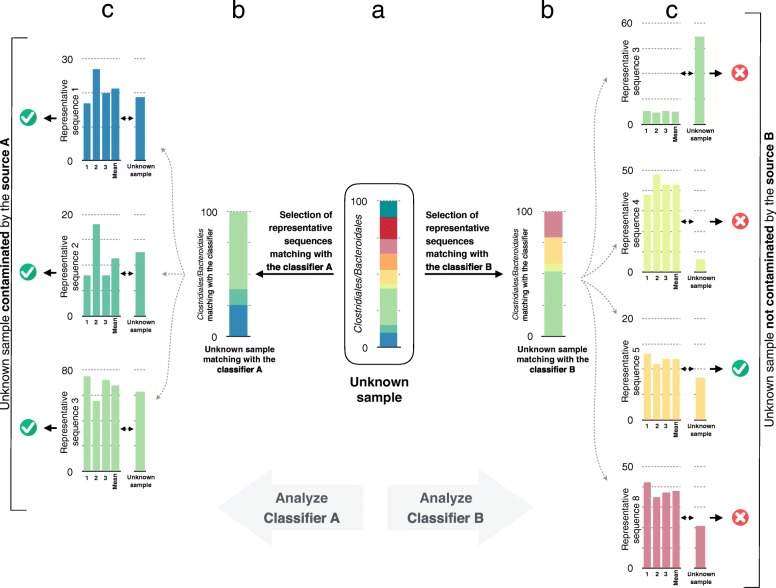
Schematic flow chart to characterize source-specific fecal contamination within an unknown environmental sample. (**a**) Determination of the bacterial profile of an environmental sample. (**b**) Selection of sequences in common with the classifier. (**c**) Comparison of the relative abundance of the selected sequences with the classifier. An unknown sample is considered contaminated by a source if the relative abundance for the majority of the selected sequences is similar to the one of the classifier

In this study, the random forest classification algorithm was used through two distinct steps. First, a classifier was built for each fecal source (Fig. 1). A classifier is composed of the representative sequences that are the most reliable to discriminate one source from another. These representative sequences are selected by running random forest classifications to compare samples from a given source to samples from all the other sources, i.e., the samples are divided into two classes: samples that belong to source X and samples that do not belong to source X. Second, for each source, the random forest algorithm is trained with the respective representative sequences selected in the first step. To classify an unknown sample, only the sequences matching the representative sequences used in a classifier are selected and their relative abundance is calculated. For each classifier, two outputs are possible. An unknown sample can be classified as “contaminated by source X” or “not contaminated by source X”. The prediction is assessed using the majority vote described previously. Roughly, it corresponds to the comparison of the sequence relative abundances between the classifier and the new samples (Fig. 2).

### Data collection

A total of 82 animal fecal samples, including 10 cats, 17 cows, 19 dogs, 11 deer, 16 pigs, 2 geese, 3 rabbits, and 4 raccoons, were collected between 2008 and 2016 in the USA, which included 2 cats from Australia. Samples were transported in sterile tubes and stored at − 80 °C until DNA extraction. To characterize human fecal contamination, we used 17 sewage influent samples from seven cities in different states in the USA collected in a former survey between 2012 and 2013 [31], and three sewage influent samples from Reus in Spain [32] and Salvador in Brazil [33]. Finally, DNA extracted from 25 freshwater samples collected in stormwater discharges, rivers, and Lake Michigan were used in classifier analyses [34, 35]. More details of sampling collection methods are reported in Additional file 1.

### Fecal indicator bacteria enumeration

Densities of fecal indicator bacteria were evaluated in freshwater samples using the USEPA standard methods 1603 and 1600 for *Escherichia coli* (*E*. *coli*) and enterococci, respectively [36, 37]. For each sample, between 1 and 100 mL of water were filtered through a 0.45-μm pore size nitrocellulose filter (Millipore, Billerica, MA). Filters were incubated for 24 h on modified membrane-thermotolerant *E*. *coli* or membrane *Enterococcus* indoxyl-d-glucoside agar plates (Becton Dickson, USA).

### Sample processing and DNA extraction

For fecal samples, bacterial DNA was extracted from approximately 0.2 g of material using QIAmp DNA stool mini kit according to the manufacturer’s instructions (Qiagen, USA). A total of 25 mL for sewage influent and 200 or 400 mL for freshwater samples were filtered onto 0.22-μm mixed cellulose ester filters with a 47 mm diameter (Millipore, USA). DNA from filters was then extracted using the FastDNA spin kit for soil (MP Biomedicals, USA) according to the manufacturer’s instructions. One modification of this protocol was applied: Cells were mechanically lysed using a MiniBeadBeater-8 cell disruptor (BioSpec Products, USA) for 1 min and 2 min at room temperature for sewage and freshwater samples, respectively. DNA was stored at − 20 °C until it was analyzed. DNA concentration was determined using a NanoDrop spectrophotometer (Thermo Fisher Scientific, USA).

### Real-time quantitative PCR analysis

Three human-associated bacterial qPCR assays were performed on freshwater samples: sewage *Lachnospiraceae*, i.e., Lachno2 [38] and Lachno3 [39] and human *Bacteroides* combining the HF183F forward primer [40] with the reverse primer and probe from Kildare et al. [41]. Freshwater samples were quantified using a StepOne Plus™ Real-Time PCR System Thermal Cycling Block using Taqman hydrolysis probe chemistry with 2X Taqman® Gene Expression Master Mix (Applied Biosystems; Foster City, CA). For each run, triplicate standard curves were generated using a linearized plasmid containing the target sequence. The slope of the standard curves varied between − 3.312 and − 3.404 for Lachno2, − 3.394 and − 3.457 for Lachno3, and − 3.195 and − 3.369 for human *Bacteroides.* A correlation coefficient higher than 0.995 was observed for each assay standard curve. Amplification efficiencies ranged from 94.66 to 105.59%. Each method had limit of quantification of 15 gene copies per reaction.

### 16S rRNA gene sequencing and library construction

Amplicon libraries were constructed at the Josephine Bay Paul Center at the Marine Biological Laboratory (Woods Hole, MA, USA) using the MiSeq Illumina® platform for the V4 to V5 hypervariable region and HiSeq or NextSeq Illumina® platforms for the V6 hypervariable region. Details for amplicon library construction and sequencing procedures for the V4 to V5 regions are described in Morrison et al. [42], and for the V6 region in Eren et al. [43]. Reads were trimmed using cutadapt v1.14 [44], allowing for four mismatches in the primer sequence. Forward and reverse reads were merged using PEAR v0.9.10 [45] using the default parameters. Using Mothur 1.39.5 [46], assembled reads were discarded if they contained ambiguous bases, had more than eight successive homopolymers, or had a length smaller/higher than 5% of the V4V5 median (372 bp) and 10% of the V6 median (60 bp). Sequences were taxonomically assigned based on the best match in a Global Alignment for Sequence Taxonomy (GAST) process [47] and the 2013 release Greengenes database [48, 49]. Only sequences assigned to *Clostridiales* and *Bacteroidales* were selected for further analysis.

A minimum entropy decomposition (MED) analysis was performed for each bacterial group assemblage using the oligotyping pipeline version 2.1 [50]. MED uses nucleotide entropy (nucleotide variant variability) to distinguish along DNA sequence differences in nucleotides originated from true genetic variation among organisms from noise due to sequencing errors. MED partitions DNA sequences into amplicon sequence variants (ASVs) according to the position in the DNA sequence with the highest entropy. This step iteratively lasts until each final ASV satisfies the maximum entropy criterion. ASVs that do not meet the minimum substantive abundance (M) criterion were discarded. M was set to *N*/10,000 for V4V5 and *N*/50,000 for V6, where *N* is the total number of sequences in the dataset.

### Random forest classifications

To create the classifiers, 100 random forests constituted of 10,000 trees were computed using the default settings of the “randomForest” function implemented in the randomForest R package [51]. Mean decrease Gini values were averaged for each ASV among the 100 random forest replicates. The ASVs with the first 200 highest mean decrease Gini values were plotted in a scree plot. ASVs with mean decrease Gini values above the breakpoint curve were chosen to be part of the classifier. Breakpoints were estimated using the “breakpoints” function included in the strucchange R package [52]. Then, the relative abundance of the selected ASVs was re-calculated. To classify unknown samples, random forest algorithm was first trained using the re-calculated relative abundance. For that, 100 random forest replicates of 1000 trees each were performed using the default settings of the "randomForest" function. Replicates were then pooled using the “combine” function. Classification of unknown samples was assessed by extracting the probability of the voting trees using the “predict” function on trimmed and merged sequences matching the classifiers’ ASVs. For the ASVs not detected in the unknown sample, a relative abundance of zero was settled.

For each unknown sample, a proxy estimating the contribution of contamination of the different sources was assessed by calculating the proportion of sequences that belong to a given classifier among the total number of sequences from all classifiers.

#### Classification of animal fecal and sewage samples

Seventy-six animal fecal and sewage samples were used to build the V6 classifiers. The predictions were tested on 23 test samples from varied animal fecal material and sewage influent.

#### Classification of contamination in freshwater samples

Freshwater samples were classified using random forest algorithm as described above. A total of 25 samples were used for V6 classifier assessment.

#### Classification of artificial bacterial assemblages

The sensitivity of random forest classifications was evaluated using artificial bacterial assemblages generated in silico*.* Defined sequence proportions from fecal and environmental samples that were not used to train the model were combined to create a matrix of artificial bacterial community mixes (Additional file 2). Sequences from each sample were selected by randomly subsampling (99 repeats), using the “rrarefy” function included in the vegan R package [53], the entire bacterial community to the desired total sequence count needed for the artificial community mixes. A freshwater sample, where the fecal indicator bacteria were not detected, was used to generate the artificial community. For example, to generate the artificial sample 1, we mixed 1% of fecal sequences from cow and deer, 5% from sewage, and 93% from freshwater samples. Fecal source sequences in these in silico generated mixes ranged from 0.01 to 20% of the total sequence count.

#### V4V5–V6 classification comparison

Performance of the classifiers was also tested on V4V5 region of the 16S rRNA gene amplicons and compared with the V6 classifier outputs. To make a direct comparison, classifiers for V4V5 and V6 were built independently using 33 animal fecal and sewage samples sequenced in both regions. Prediction accuracy was evaluated using an additional 12 test samples sequenced in both regions (see Additional file 1 for more details).

All analyses were conducted using the statistical environment R version 3.3.2 [54].

### Robustness of the predictions

Accuracy and robustness of the random forest classifications were estimated by repeating the training and the prediction of the “unknown samples” steps 100 times and computing the mean and the standard deviation of voting tree probabilities.

## Results

### Development and attributes of the V6 classifiers

Six fecal sources were used to create the classifiers, including samples from 9 cats, 15 cows, 9 deer, 15 dogs, 14 pigs, and 14 sewage samples to represent humans. Overall, 48% of the V6 sequences were assigned to *Clostridiales* and 35% to *Bacteroidales* in the animal fecal samples. In sewage samples, *Clostridiales* and *Bacteroidales* represented on average of 12% and 13% of the total sequences, respectively. For both orders, the MED analysis retained 90% of the total sequences, i.e., 21,965,364 *Clostridiales* sequences and 15,900,401 *Bacteroidales* sequences. These sequences were clustered into 2724 amplicon sequence variants (ASVs) for *Clostridiales* and 1479 ASVs for *Bacteroidales*. The bacterial assemblage for both bacterial groups was more consistent in the sewage samples than in the animal fecal samples (Bray–Curtis (BC) dissimilarity index, *Clostridiales*: BC_animal(intra sources)_ = 69 ± 19%, BC_sewage_ = 29 ± 7%; *Bacteroidales*: BC_animal(intra sources)_ = 74 ± 23%, BC_sewage_ = 37 ± 15%, Additional file 3).

**Fig. 3:**
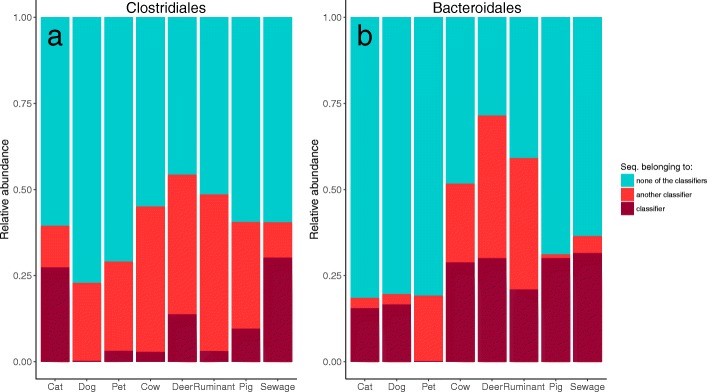
Distribution of the sequences within the bacterial groups. Relative abundance per source of the sequences belonging to the respective source classifier (dark red), to another classifier (light red) or not belonging to any of the classifiers (blue) for the (**a**) *Clostridiales* and (**b**) *Bacteroidales* groups

**Fig. 4:**
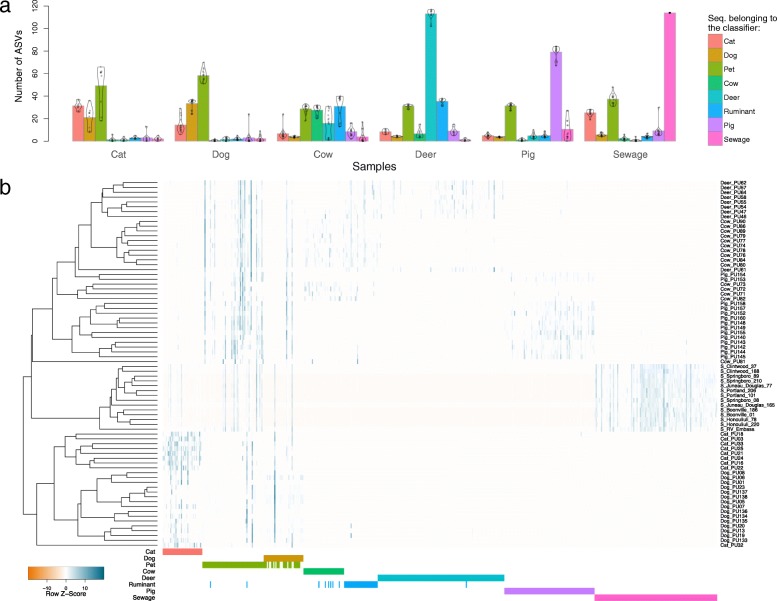
Distribution of amplicon sequence variants (ASVs) selected among the *Clostridiales* classifiers. (**a**) Mean and distribution of the number of ASVs belonging to the different classifiers for each source of fecal samples. (**b**) Heatmap representing the relative abundance of the ASVs selected within the eight classifiers (represented on the horizontal axis) within the samples (listed on the right) used to build the classifiers. Samples were clustered using the UPGMA algorithm based on the Bray–Curtis dissimilarity matrix

One classifier was built for each source by selecting the sequences with the highest mean decrease in Gini values. In addition to the six sources investigated, a “Pet” and a “Ruminant” classifier were built by merging the Cat/Dog, and Cow/Deer samples, respectively. These two extra sources were created after preliminary investigations, which revealed high out-of-bag error rate within Cat, Dog, Cow and Deer samples, resulting from shared ASVs between sources. About the same number of ASVs were selected within the classifiers for both bacterial groups, with an average of 69 and 55 ASVs for *Clostridiales* and *Bacteroidales*, respectively (Additional file 4). Overall, ~ 17% of the total sequences within each bacterial group comprised the classifiers (Fig. 3). However, for the animal sources, the proportion of sequences belonging to the different classifiers was higher for *Bacteroidales* compared with *Clostridiales* (Fig. 3). In addition, a relatively low proportion (30%) of the unique ASVs comprising the classifiers were found exclusively in a single fecal source, and these were at low abundance, except for the Cat–*Clostridiales*/*Bacteroidales* and Dog–*Clostridiales* classifiers, which had no exclusive ASVs (Additional file 4). This result indicates that a large proportion of ASVs selected in the classifiers were source-preferred (Fig. 4a), i.e., ASVs were common to multiple sources, but with distinct abundance patterns for specific sources. Differences between sources in the assemblage of the ASVs selected in the classifiers are visualized on Fig. 4b and Additional file 5. Despite the intra-source variability, distinctive inter-source patterns allowed for discrimination of sample sources.

### Classifications of animal fecal and sewage samples

The specificity of predictions was evaluated on 14 animal and sewage samples collected from different locations, and not used to create the V6 classifiers (Additional file 1). Overall, predictions obtained from *Clostridiales* and *Bacteroidales* classifiers were comparable (Table 1). Both bacterial order classifiers correctly identified the fecal signature in cow, deer, pet, pig, ruminant, and sewage. Misclassifications occurred for the *Bacteroidales* classifiers on samples Cat_PU15 and Dog_PU17, but when considered as the pet group, they were correctly classified. The proportion of ASVs matching with each source classifier within the test samples was significantly higher for the *Bacteroidales* classifiers than the *Clostridiales* classifiers (Mann–Whitney, *W* = 228.5, *n*_Clo._ = 18, *n*_Bac_ = 16, *P* = 0.004), suggesting more shared signature sequences in *Bacteroidales*.

**Table 1:** Prediction of the fecal source contamination for animal fecal and sewage samples

Unknown sample ID	*Clostridiales*	*Bacteroidales*
Cat_PU15	Cat^a^ (88)–Pet^a^ (100)	Pet^a^ (90)
Cow_PU75	Cow^a^ (68)–Ruminant^a^ (12)	Cow^a^ (93)–Ruminant^a^ (97)
Deer_PU11	Deer^a^ (93)–Ruminant^a^ (16)	Deer^a^ (100)–Ruminant^a^ (100)
Dog_PU12	Dog^a^ (22)–Pet^a^ (100)	Dog^a^ (64)–Pet^a^ (100)
Dog_PU17	Dog^a^ (86)–Pet^a^ (100)	Cat^a^ (7)–Pet^a^ (99)
Pig_PU156	Pig^a^ (48)	Pig^a^ (99)
Pig_PU159	Pig^a^ (46)	Pig^a^ (99)
Cow_PU70&Deer_PU91	Cow^a^ (38)–Deer^a^ (29)–Ruminant^a^ (10)	Cow^a^ (88)–Deer^a^ (12)–Ruminant^a^ (100)
Sewage_Duncansville_161	Sewage^a^ (83)	Sewage^a^ (93)
Sewage_Duncansville_52	Sewage^a^ (86)	Sewage^a^ (97)
Sewage_Milwaukee_JI199	Sewage^a^ (85)	Sewage^a^ (94)
Sewage_Milwaukee_SS200	Sewage^a^ (87)	Sewage^a^ (96)
Sewage_ReusSpain_224	Sewage^a^ (88)	Sewage^a^ (99)
Sewage_ReusSpain_80	Sewage^a^ (95)	Sewage^a^ (99)
OtherSource_Goose_PU126	–	–
OtherSource_Goose_PU97	Pet^b^ (64)	–
OtherSource_Rabbit_PU26	–	–
OtherSource_Rabbit_PU27	–	–
OtherSource_Rabbit_PU9	–	–
OtherSource_Raccoon_PU100	–	Dog^a^ (85)–Pet^a^ (72)
OtherSource_Raccoon_PU101	Pet^b^ (98)	–
OtherSource_Raccoon_PU102	Dog^c^ (59)–Pet^a^ (71)	–
OtherSource_Raccoon_PU52	–	Dog^c^ (91)

Values representing the proportion of sequences that belong to a given classifier among the total number of sequences from all classifiers

^a^Index representing the percentage of the vote by the trees higher than the majority (50%)

^b^Index representing the percentage of the vote by the trees between 45 and 50%

^c^Index representing the percentage of the vote by the trees between 40 and 45%

The specificity of predictions was also tested on nine animal fecal samples from hosts not used as classifier sources, i.e., goose, rabbit, and raccoon. In general, samples were not classified to the eight fecal sources studied (Table 1). Five samples were misclassified as a “Dog” and/or “Pet” by either *Clostridiales* and *Bacteroidales* classifiers, but not by both bacterial group classifiers at the same time.

### Classification of environmental samples

The accuracy of predictions of human fecal contamination using random forest classification was assessed using 25 freshwater samples, 14 of which had evidence of sewage contamination based on qPCR detection of human-associated fecal indicators (Table 2). Among these 14 contaminated freshwater samples, *Clostridiales* and *Bacteroidales* classifiers identified a human bacterial signature in 10 and 12 samples, respectively. Nine samples (64%) were classified correctly by both bacterial group classifiers. No sample was classified with fecal pollution from a non-human source.

**Table 2:** Random forest classification of 25 freshwater samples with different level of fecal contamination

Random forest classifications^†^						
Environmental sample ID	Type of sample	Major type of contamination	Level of fecal indicator bacteria^‡^	Level of qPCR human marker^‡‡^	*Clostridiales*	*Bacteroidales*
FMRMN73_092	Stormwater	HC	High	High	Sewage^a^ (98)	Sewage^a^ (99)
FMRMN73_29	Stormwater	HC	High	High	Sewage^a^ (84)	Sewage^a^ (89)
FMRHC33_42	Stormwater	HC	High	Medium	–	Sewage^c^ (100)
FMRMN60_100	Stormwater	HC	High	High	–	–
FMRMN29_108	Stormwater	HC	High	High	Sewage^a^ (91)	Sewage^a^ (95)
MKE_162	River	HC	High	Medium	Sewage^b^ (85)	Sewage^a^ (98)
MNE_163	River	HC	Medium	Medium	Sewage^b^ (57)	Sewage^b^ (86)
KK_160	River	HC	Medium	High	Sewage^c^ (77)	Sewage^a^ (99)
MNE_159	River	HC	Medium	Medium	Sewage^c^ (68)	Sewage^b^ (98)
MKE_158	River	HC	Medium	Medium	–	Sewage^b^ (98)
Gap_51	Harbor	HC	Medium	High	Sewage^a^ (82)	Sewage^a^ (97)
Junction_54	Harbor	HC	Low	Medium	–	Sewage^b^ (78)
Gap_55	Harbor	HC	Low	Medium	Sewage^a^ (55)	Sewage^c^ (94)
Junction_52	Harbor	HC	Low	Medium	Sewage^c^ (64)	–
FMRMN53_26	Stormwater	NHC	High	Inconclusive	–	–
SHC12A_10	Stormwater	NHC	High	Inconclusive	Sewage^c^ (90)	–
SMN17A_20	Stormwater	NHC	High	Inconclusive	–	Sewage^c^ (100)
FMRHC43_43	Stormwater	NHC	High	Not detected	–	–
FMRHAC22_38	Stormwater	NHC	Medium	Not detected	–	–
Gap_53	Harbor	NHC	Low	Not detected	–	Sewage^b^ (99)
1_mile	Lake	NC	Not detected	Not tested	–	–
2_miles	Lake	NC	Not detected	Not tested	–	–
DocIn_155	Lake	NC	Not detected	Not tested	–	–
DocMid_156	Lake	NC	Not detected	Not tested	–	–
DocOut_157	Lake	NC	Not detected	Not tested	–	–

*HC* human contamination (fecal indicator bacteria and human marker detected), *NHC* non-human contamination (fecal indicator detected and human markers not detected or inconclusive reflecting potential for low levels of human contamination), *NC* not fecal contaminated (fecal indicator not detected)

^†^Values in parentheses represent the proportion of sequences that belong to a given classifier among the total number of sequences from all classifiers

^‡^Density levels of the fecal indicator *E*. *coli* and enterococci: not detected, 0; low, > 0–250; medium, 250–1000; high, > 1000 CFU/100 mL

^‡‡^Quantification levels of the markers human *Bacteroides*, Lachno2, and Lachno3 when tested: Not detected, 0; not quantifiable, > 0–15; low, > 15–100; medium, 100–10,000; high, > 10,000 gene copies/100 mL. In case of divergence between the human *Bacteroides*, Lachno2, and/or Lachno3 human markers, results were considered to be inconclusive. See Additional file 1 for details

^a^Index representing the percentage of the vote by the trees higher than the majority (50%)

^b^Index representing the percentage of the vote by the trees between 45 and 50%

^c^Index representing the percentage of the vote by the trees between 40 and 45%

The samples classified as human-contaminated shared at least 39 and 45% of their unique sequences with *Clostridiales* and *Bacteroidales* sewage classifiers, respectively, while for example, the unclassified sample FMRMN60_100, with evidence of human contamination by qPCR, shared only 31 and 40% of its unique sequences with the respective bacterial groups. Other stormwater samples with high levels of *E*. *coli* and enterococci, but low or inconclusive qPCR human markers, likely contained primarily non-human fecal contamination but were potentially mixed with low levels of sewage. In these cases, classification using random forest was also inconclusive. The majority of river and harbor samples with medium and high levels of human qPCR markers were correctly classified as having sewage contamination, with exceptions being cases where only one of the classifiers (*Clostridiales* or *Bacteroidales)* indicated the presence of sewage. Although Gap_53 had a low level of fecal contamination, with no evidence of human contamination using qPCR markers, the *Bacteroidales* classifier identified a sewage signature. All freshwater samples with no evidence of fecal pollution were correctly determined to have none of the examined fecal sources.

### Classification of the artificial bacterial assemblage

**Fig. 5:**
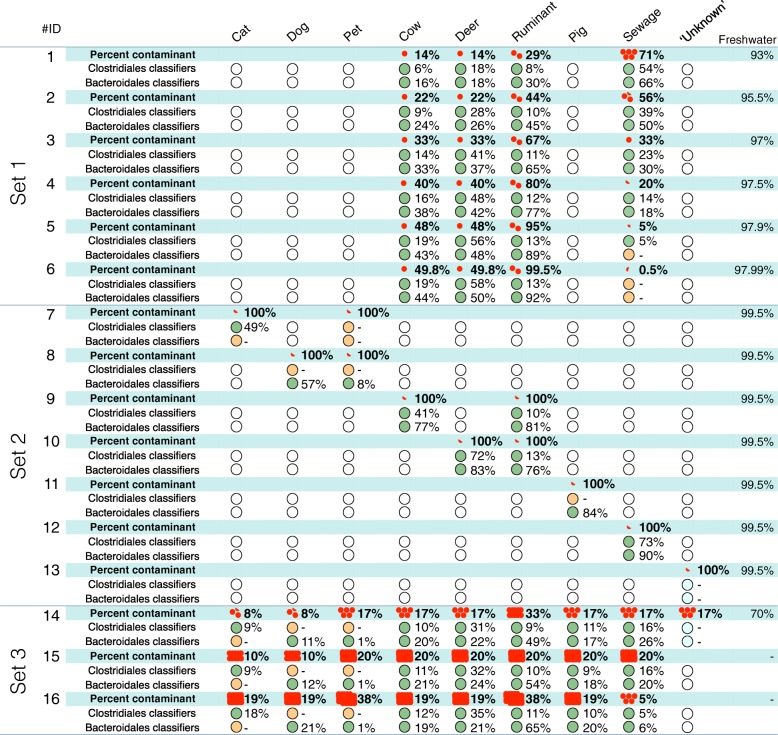
Random forest classifications performed on 16 artificial bacterial assemblages generated in silico. Red dots show the proportion of sequences within the total assemblage belonging to a source (1 dot = 1%). The percentage listed in the freshwater column corresponds to the proportion of sequences from the non-contaminated freshwater sample within the total assemblages (per sample, red dots + freshwater percentages = 100%). Bold values associated with red dots indicate the proportion of contamination expected for the different sources. Predictions of the *Clostridiales* and *Bacteroidales* classifications are indicated in the white rows. Green circles indicate the classifier detected the source signature in the sample. Orange circles indicate the classifier did not detect the source signature when it was expected. Blue circles indicate the classifier did not detect a signature when a source not included in the classifiers was included in the assemblage. The proportion of sequences matching the source classifier is associated with the green circles. See Additional file 2 for more details

To evaluate the sensitivity and accuracy of random forest classifications, 37 in silico artificial bacterial assemblages were generated (Fig. 5 and Additional file 2). These tests indicate (set 1, Fig. 5) that both bacterial group classifiers could detect the sewage signature in complex samples with animal sources when at least 0.5% of the total bacterial assemblage was composed of sewage sequences; in this case, 0.05% of the total sequences match the sewage classifiers (Additional file 2). Further testing of contamination levels, i.e., 1, 0.5, 0.1, and 0.01% of sequences, from each of the animal fecal sources, revealed similar sensitivity thresholds, that is, sources were correctly classified when sequences from the fecal source comprised 0.5% of total assemblage (set 2, Fig. 5) and approximately 0.001 to 0.06% of sequences in the total assemblage matched the classifiers (set 2 Additional file 2). The Cat_PU15, Dog_PU12, and Pig_159 samples did not follow this trend. In set 3 of the in silico samples, we found the classifiers could identify many contamination sources simultaneously when the sequences from these sources were pooled (Fig. 5). Across all tests, the “Cat”, “Dog”, and “Pet” signatures were the most difficult to predict (lowest accuracy) and generally required higher levels of contamination (i.e. > 10% of source sequences present in the artificial assemblages) for accurate contamination prediction.

For sets 1 to 4 (Fig. 5 and Additional file 2), the expected proportion of source contaminations were correlated significantly with the relative proportion of sequences matching the classifiers among sequences belonging to all classifiers. Therefore, these proportions could be used as a proxy for the relative magnitude of contamination from individual sources. This correlation was stronger for *Bacteroidales* (Spearman’s rank correlation coefficient Rs = 0. 790, *n* = 59, *P* < 0.001) than *Clostridiales* (Rs = 0. 546, *n* = 54, *P* < 0.001). The proxy we developed could not estimate the proportion of fecal contamination from sources that were not used to build the classifier (e.g., see Fig. 5 tests 13 and 14).

### Comparison of the V4V5–V6 classifications

Random forest classifiers were also built and tested using the V4V5 hypervariable region of the 16S rRNA gene. Predictions were compared with new V6 classifiers created from a subset of the original sample set and with the same samples used to generate the V4V5 classifiers. Characteristics of the classifiers are detailed in Additional file 4. Both V4V5 and V6 classifiers accurately identified most of the fecal signatures (Table 3). However, as observed with the V6 classifier built with a more extensive sample set, some misclassifications were detected for the “Cat” and “Dog” sources. Rabbit_PU26, a source that was not present in any classifier, was correctly not associated with a fecal source except for the V4V5 *Clostridiales* classifier, in which it was classified as a Pet source. Both bacterial group classifiers estimated comparable proportions of source contamination between the V4V5 and V6 regions (Mann–Whitney, *Clostridiales*: *W* = 120, *n* = 27, *P* = 0.159; *Bacteroidales*: *W* = 96, *n* = 24, *P* = 0.173).

**Table 3:** V4V5 and V6 classifier predictions for animal fecal, sewage, and freshwater samples

Unknown sample ID	V4V5 region		V6 region	
	*Clostridiales*	*Bacteroidales*	*Clostridiales*	*Bacteroidales*
Cat_PU15	Pet^a^ (9)	Cat^c^ (86)–Pet^a^ (84)	Cat^a^ (70)–Pet^a^ (72)	Pet^a^ (1)
Cow_PU75	Cow^a^ (29)–Ruminant^a^ (99)	Cow^a^ (88)–Ruminant^a^ (97)	Cow^a^ (21)–Ruminant^a^ (53)	Cow^a^ (76)–Ruminant^a^ (97)
Deer_PU11	Deer^a^ (59)–Ruminant^a^ (92)	Deer^a^ (100)–Ruminant^a^ (100)	Deer^a^ (36)–Ruminant^a^ (52)	Deer^a^ (93)–Ruminant^a^ (100)
Dog_PU17	Dog^a^ (65)–Pet^a^ (46)	–	Dog^a^ (94)–Pet^a^ (91)	Cat^a^ (5)–Pet^a^ (56)
Pig_PU159	Pig^a^ (89)	Pig^a^ (97)	Pig^a^ (27)	Pig^a^ (96)
Cow_PU70&Deer_PU91	Ruminant^a^ (99)	Cow^a^ (77)–Deer^c^ (20)–Ruminant^a^ (96)	Cow^a^ (10)–Deer^c^ (5)–Ruminant^a^ (44)	Cow^a^ (67)–Ruminant^a^ (100)
Sewage_Duncansville_52	Sewage^a^ (94)	Sewage^a^ (90)	Sewage^a^ (89)	Sewage^a^ (86)
Sewage_MilwaukeeJI_199	Sewage^a^ (93)	Sewage^a^ (76)	Sewage^a^ (88)	Sewage^a^ (68)
Sewage_MilwaukeeSS_200	Sewage^a^ (94)	Sewage^a^ (81)	Sewage^a^ (87)	Sewage^a^ (80)
Sewage_ReusSpain_224	Sewage^b^ (96)	Sewage^b^ (99)	Sewage^a^ (86)	Sewage^a^ (97)
Sewage_ReusSpain_80	Sewage^a^ (97)	Sewage^b^ (99)	Sewage^a^ (91)	Sewage^a^ (95)
OtherSource_Rabbit_PU26	Pet^b^ (75)	–	–	–

Values in parentheses represent the proportion of sequences that belong to a given classifier among the total number of sequences from all classifiers

^a^Index representing the percentage of the vote by the trees higher than the majority (50%)

^b^Index representing the percentage of the vote by the trees between 45 and 50%

^c^Index representing the percentage of the vote by the trees between 40 and 45%

### Robustness of predictions

The robustness of the predictions, i.e., the probability generated by random forest voting trees, was assessed by repeating 100 times the random forest training and classifications of all animal, sewage, and freshwater samples described above. For the V6 analysis, the results indicated good reproducibility of the predictions, with an average standard deviation across the trials of 0.05% and 0.04% for *Clostridiales* and *Bacteroidales*, respectively, and a maximum of 0.18%. No significant difference in prediction robustness was observed between the two bacterial groups (Mann–Whitney, *W* = 74,420, *n* = 768, *P* = 0.821). Similar standard deviation values were obtained for the V4V5–V6 comparison classifiers, with an average of 0.06% for both V4V5 and V6 classifiers (maximum_V4V5_ = 0.17%, maximum_V6_ = 0.16%). No significant difference was observed between the two regions (Mann–Whitney, *W* = 18,384, *n* = 385, *P* = 0.965).

## Discussion

### *Clostridiales* and *Bacteroidales* community assemblages provide a signature of sewage and animal fecal contamination

Despite the incredible diversity within the microbial world, evolutionary forces predominantly favored members of the *Clostridiales* and *Bacteroidales* orders in the gut of animals and humans (see reviews [2, 55]). Within each of these fecal bacterial group assemblages, we observed reliable patterns that discriminated host sources. This signal was preserved despite variation among individual animals. Further, host patterns were observed across multiple taxonomic levels: While our respective classifiers included sequences from either *Clostridiales* or *Bacteroidales*, preliminary investigations targeting *Bacteroidaceae*, *Lachnospiraceae*, *Prevotellaceae*, or *Ruminococcaceae* individual family level classifiers showed relevant bacterial patterns between hosts (Additional file 6). Similar observations were also found when focusing solely on the genus *Blautia* [5]. Taken together, these results highlight the fractal nature of gut microbial communities, i.e., similar patterns at increasingly smaller scales of the gut microbiome. The community differentiation among hosts stems from (i) traits conserved across broad taxonomic groups and (ii) selection within closely related microbial genera/species for members that are specialized for a particular host niche [4, 56, 57].

In this work, we focused on *Clostridiales* and *Bacteroidales* and ignored the remaining community data. This taxonomically narrow focus on the two most common gut-associated groups removes the influence of large cross-phylum shifts in the bacterial community while providing fecal source identification redundancy. Host diet and/or transient bacteria can cause changes in gut microbiota composition that are not typical of the host species in general [58, 59]. For example, the ingestion of *Lactobacillus* strains can increase temporarily their recovery in fecal samples or lead to phylum-level dominance shifts in the community (see review and references therein [58]). The concept that population shifts in response to environmental gradients are standardly assessed within species has been reported previously, including in macro ecology [60, 61].

Major shifts in bacterial assemblages may also occur during sample processing or sequencing, where freezing, duration of storage, choice of DNA extraction method, or choice of primers inflated the recovery of certain microbial phyla leading to an important shift in bacterial phylum-level ratios, such as *Firmicutes*-to-*Bacteroidetes* abundance [62–66]. In general, it is believed that there is more consistency in sample processing recovery among more closely related bacterial groups due to their similar cell membrane properties. Overall, classifiers with a narrow taxonomic focus may be less influenced by extraneous factors.

### Random forest classifiers are representative of host groups

A relatively small number of unique ASVs, representing a moderate proportion of the *Clostridiales* and *Bacteroidales* assemblages, were selected by random forest as the bacterial signature of the different fecal sources. The classifiers contained a few unique ASVs that were exclusive to a source; most ASVs were host-preferred (70%), i.e., shared with other sources but with differential abundance patterns. This result supports our previous findings, highlighting that bacterial lineage abundances were more important than presence/absence patterns for discriminating sources [5, 35].

Random forest was effective in retrieving differential bacterial host signatures between the sources investigated, suggesting (i) an adequate number of samples were analyzed to provide a good coverage of the host group in the classifiers, and (ii) there is consistency in the bacterial signatures within each of the sources investigated. The effectiveness of random forest was reflected in the low out-of-bag error rates, which indicated the degree of coverage of the host groups. These low values were observed even by training the classifiers using on average as few as ten animal fecal/sewage samples. This result suggests that random forest is sensitive enough to identify, with a small number of samples, relevant host bacterial group patterns. However, it would be pertinent to assess the out-of-bag error rates and the accuracy of the predictions using samples collected outside the USA, since geographically related environmental factors have been observed to affect host animal fecal microbiomes, and thus source identifications [67]. Additionally, the creation of classifiers with samples collected from distinct locations could also expand and provide more geographic and host group coverage. We also note in the V4V5 and V6 classifier comparison, where less samples were used to train the classifiers, the out-of-bag error rates were higher and the number of accurate classifications was lower.

The sewage pattern was highly consistent among cities used to build the classifiers. We previously showed that sewage is an aggregate signal from human populations, and this signal does not vary much across the USA [31]. This observation appears to extend to regions outside the USA as the sewage signature was found in geographically distant samples collected in Spain. Moreover, the classifiers properly detected the human signature in a large number of environmental samples with evidence of human fecal contamination as detected by PCR-based markers. However, the sewage signal for some samples, notably stormwater samples, was not detected when sewage contamination was thought to be present based on human fecal marker assays. In these samples, the contamination may originate from a few individual humans, which presents a highly variable fecal bacterial signature compared to the integrative signature in sewage. This observation supports previous findings in which human fecal indicators detected by qPCR in stormwater samples were inconsistent [20]. The stormwater samples may be mainly contaminated by urban wildlife or other uncharacterized fecal sources. Inconclusive qPCR and random forest classifications suggest either the sources responsible for the fecal contamination share bacterial community members with humans or very low levels of human fecal pollution are present, but could not be verified. In either case, the sequence data did not resolve the inconclusive qPCR results. No other fecal animal sources were detected in the environmental samples studied. New classifiers of urban animals that contribute to runoff may be necessary to further delineate sources of fecal pollution in stormwater.

### Random forest classification: a powerful source identification tool

This study highlighted the suitability of random forest classification approach to perform fecal source identification using 16S rRNA gene amplicons. Targeting the V6 hypervariable region provided enough variability in bacterial community composition between hosts to properly classify the source of fecal contamination, with only a few false-positive pet signatures detected. Besides the high accuracy classification, random forest presents the advantage that the addition of new samples in a classifier or the creation of new source classifiers is relatively simple. Hence, random forest can be considered as a scalable and extensible model. As highlighted by Statnikov et al. [23], the creation and training of classifiers are not impacted by the presence of a few poorly sequenced samples (data not shown). Moreover, once the classifiers are created, the training of the classifiers with a subset of the data and the classification of unknown samples takes a few seconds and does not need important computing resources.

Unlike the widely used microbial source tracking tool SourceTracker [7, 16, 20], random forest classification cannot estimate the proportion of uninvestigated sources. However, the proxy we developed allowed us to estimate the proportions of fecal contamination among the sources investigated. Since these proportions are not quantitative, we suggest that they be used for hierarchical classification to identify which source(s) contributed the most to observed fecal pollution.

Our classifier approach will be applicable to sequence data from other regions of the 16S rRNA gene or other genes, as seen in the V4V5 and V6 comparison. Although the V6 region is one of the shortest commonly sequenced hypervariable regions, i.e., the least amount of sequence information, this region is associated with the highest degree of polymorphism [68]. Short read lengths make the V6 target region suitable for deep sequencing platforms such as the NextSeq Illumina platform that can generate 400 million of reads per run compared to 25 million for the MiSeq Illumina platform [69]. This difference of sequencing depth may be key for detecting low level of fecal contamination in environmental samples.

## Conclusion

We demonstrate the relevance of using random forest classification as an efficient and effective tool for source identification. The method is scalable and extensible for systematically assessing complex sequence data to identify fecal sources and provide the associated proportion of contamination. Both *Clostridiales* and *Bacteroidales* bacterial groups appear to be relevant markers of animal and sewage fecal contamination in the environment. Using both classifiers offers independent verification of contamination to increase confidence in results. Further, by allowing a fast-screening of large sequence datasets, this approach could also be useful in identifying new molecular markers for source microbiomes. High-throughput sequencing is emerging as a promising approach for water quality assessments due to the falling costs of sequencing, the exponential increase in information gained from these methods, and the development of field adaptable platforms such as MinION based on Nanopore technology [70]. The limitation in implementing such approaches may not be sequencing technology, but the need for rapid computational methods with low-resource demands. Since random forest classification does not necessitate significant computing resources, this approach represents a valuable tool for assessment of contaminated water for pathogen risk and for stakeholders to identify the main sources of fecal pollution and implement appropriate management actions.

## Additional files

Additional file 1: Sample metadata. Sample metadata includes Sample ID of the samples used to create or test the classifiers in the paper, sample origin and collection date. The volume or the weight, as well as the name of the DNA kits used to extract total bacterial DNA are also mentioned. The densities of fecal indicator bacteria and human markers in the freshwater samples are indicated. Short Read Archive and the individual SRR accession number are listed to be used to reference the raw sequence file from NCBI. (XLSX 138 kb)

Additional file 2: Random forest classifications performed on 37 artificial bacterial assemblages. This file contains (a) the classifications of 37 artificial bacterial assemblages, and (b) the number of sequences associated with the different classifiers. The file lists the sample IDs, the proportions used to generate the in silico artificial bacterial assemblages, the expected and observed fecal source contamination proportions, and the total number of sequences per sample. (XLSX 52 kb)

Additional file 3: Bray–Curtis dissimilarity matrix. Bray–Curtis dissimilarity matrix for *Clostridiales* and *Bacteroidales* bacterial composition among samples used to build the classifiers. (XLSX 133 kb)

Additional file 4: Classifiers properties. This file indicates the number of ASVs selected to compose the classifiers, the number of specific ASVs and the out-of-bag error rate per source for the V6 study and the V4V5 to V6 comparison analysis. (XLSX 25 kb)

Additional file 5: Distribution of the ASVs selected among the *Bacteroidales* classifiers. This file presents (a) the mean and distribution of the number of ASVs belonging to the different classifiers for each source of fecal samples, and (b) a heatmap representing the relative abundance of the ASVs selected within the eight classifiers for the samples used to build the classifiers. (DOCX 4000 kb)

Additional file 6: Random forest predictions for the fecal-animal, sewage and freshwater samples using V6 classifiers built at the family level. This file contains the classifications of 48 samples using *Bacteroidaceae*, *Lachnospiraceae*, *Prevotellaceae* and *Ruminococcaceae* classifiers. (XLSX 29 kb)

## Abbreviations

ASV: Amplicon sequence variant
BC: Bray–Curtis
DNA: Deoxyribonucleic acid
HC: Human contaminated
MDG: Mean decrease Gini
MED: Minimum entropy decomposition
NC: Not contaminated
NHC: Not human contaminated
PCR: Polymerase chain reaction
qPCR: Real-time quantitative PCR
rRNA: Ribosomal ribonucleic acid
