# Supplementary material for: Fecal source identification using random forest
Source: Microbiome. 2018 Oct 18;6:185. doi: 10.1186/s40168-018-0568-3 (PMC6194674; doi:10.1186/s40168-018-0568-3)
Supplement: Supplementary file 5 — Distribution of the ASVs selected among the Bacteroidales classifiers. This file presents (a) the mean and distribution of the number of ASVs belonging to the different classifiers for each source of fecal samples, and (b) a heatmap representing the relative abundance of the ASVs selected within the eight classifiers for the samples used to build the classifiers. (DOCX 4000 kb) [file 40168_2018_568_MOESM5_ESM.docx]

**Additional file 5 Distribution of the ASVs selected among the *Bacteroidales* classifiers***(a) Mean and distribution of the number of ASVs belonging to the different classifiers for each source of fecal samples. (b) Heatmap representing the relative abundance of the ASVs selected within the eight classifiers (represented on the horizontal axis) within the samples (listed on the right) used to build the classifiers. Samples were clustered using the UPGMA algorithm based on the Bray–Curtis dissimilarity matrix.*
